# Principal contribution of *HLA-DQ* alleles, DQB1*06:04 and DQB1*03:01, to disease resistance against primary biliary cholangitis in a Japanese population

**DOI:** 10.1038/s41598-017-11148-6

**Published:** 2017-09-11

**Authors:** Michio Yasunami, Hitomi Nakamura, Katsushi Tokunaga, Minae Kawashima, Nao Nishida, Yuki Hitomi, Minoru Nakamura

**Affiliations:** 1 Department of Medical Genomics, Life Science Institute, Saga-Ken Medical Centre Koseikan, Saga, 840-8571 Japan; 2 Department of Clinical Medicine, Institute of Tropical Medicine, Nagasaki University, Nagasaki, 852-8523 Japan; 3 Clinical Research Center, National Hospital Organization (NHO) Nagasaki Medical Center and Department of Hepatology, Nagasaki University Graduate School of Biomedical Sciences, Omura, 856-8562 Japan; 4 Department of Human Genetics, Graduate School of Medicine, the University of Tokyo, Tokyo, 113-0033 Japan; 5 The Research Center for Hepatitis and Immunology, National Center for Global Health and Medicine, Ichikawa, 272-8516 Japan; 6 Headquarters of PBC Research in the NHO Study Group for Liver Disease in Japan (NHOSLJ), Omura, 856-8562 Japan; 7 Headquarters of gp210 Working Group in Intractable Hepatobiliary Disease Study Group supported by the Ministry of Health, Labour and Welfare of Japan (gp210WG), Omura, 856-8562 Japan

## Abstract

Identification of the primary allele(s) in *HLA class II* associated diseases remains challenging because of a tight linkage between alleles of *HLA-DR* and *-DQ* loci. In the present study, we determined the genotypes of seven *HLA* loci (*HLA-A*, *-B*, *-DRB1*, *-DQA1*, *-DQB1*, *-DPA1* and *-DPB1*) for 1200 Japanese patients with primary biliary cholangitis and 1196 controls. Observation of recombination derivatives facilitated an evaluation of the effects of individual *HLA* alleles consisting of disease-prone/disease-resistant *HLA* haplotypes. Consequently, a primary contribution of DQB1*06:04 (odds ratio: 0.19, p = 1.91 × 10^−22^), DQB1*03:01 (odds ratio: 0.50, p = 6.76 × 10^−10^), DRB1*08:03 (odds ratio: 1.75, p = 1.01 × 10^−7^) and DQB1*04:01 (odds ratio: 1.50, p = 9.20 × 10^−6^) was suggested. Epistasis of the protective DQB1*06:04 to risk conferred by DRB1*08:03 was demonstrated by subpopulation analysis, implicating the presence of an active immunological mechanism that alleviates pathogenic autoimmune reactions. Further, the contribution of the aforementioned *HLA* alleles as well as an *HLA-DP* allele, DPB1*02:01 to the association signals of 304 loci among 4103 SNPs in the *HLA* region at the genome-wide level of significance (p values less than 5 × 10^−8^) was demonstrated by the stepwise exclusion of the individuals possessing these *HLA* alleles from the comparison.

## Introduction

Primary biliary cholangitis (PBC) is a relatively rare disease that is predominantly observed in middle-age women. It is characterized by chronic and progressive destruction of intra-hepatic bile ducts and cholestasis^1^. Several lines of evidence have demonstrated that autoimmunity contributes to the development of PBC, as a consequence of the breakdown of immunological tolerance to autologous antigens. These include pathognomonic antibodies against mitochondrial components (anti-mitochondrial antibodies, AMA) produced very early in the disease process^2^, the involvement of cell-mediated immunity as suggested by histological findings showing the accumulation and activation of immune competent cells in the portal area of the liver and cellular responses to autologous antigens in *in vitro* studies^3–5^, and the fact that patients often experience a wide spectrum of autoimmune disorders such as Sjögren’s syndrome, autoimmune hepatitis, Hashimoto’s thyroiditis, rheumatoid arthritis, and systemic sclerosis (including limited cutaneous systemic sclerosis, formerly known as CREST syndrome)^6, 7^. As is the case in other autoimmune disorders, PBC has been associated with *HLA* polymorphisms^8–13^, and in most of these conditions, the impact of specific *HLA* alleles on the antigenic repertoire of effector cells was suggested as a mechanism underlying autoimmunity.

Genome-wide association studies (GWAS) of PBC in different populations have revealed the involvement of genetically determined alterations in certain immunological pathways, such as those related to IL12 signal transduction, TNF/TLR signal transduction, and B cell differentiation to plasma cells. However, most of these genes have not been universally identified in studies thus far, with the exception of polymorphic markers in the *HLA* region^14–16^. One of the outstanding features of *HLA* genes is that they exhibit the highest degree of polymorphism among human functional genes. Hundreds to thousands of alleles have been identified at the loci encoding HLA class I (HLA-A, -B, and -C) and class II (HLA-DR, -DQ, and -DP) molecules, some of which exist in particular preferential combinations known as “common *HLA* haplotypes” in a relatively ethnicity-specific manner. In the present study, we examined the effects of *HLA* polymorphisms on the development of PBC and demonstrated that multiple *HLA* alleles show highly significant genome wide-association signals for single-nucleotide polymorphisms (SNPs) in the *HLA* region.

## Results

### Clinical characteristics of the study population

This study enrolled 1200 Japanese patients with PBC (Table 1). A female predominance was observed, with a female to male ratio of 7.63. The majority of patients (71.6%) did not progress beyond clinical stage I by the time of their latest clinical evaluation. Patients in the clinical stage III group included 112 cases who had undergone liver transplantation (9.4%) and 8 cases who died of progression to hepatic failure (0.7%). Clinical and histological staging did not differ between genders.

**Table 1:** Basic characteristics of the patients with PBC.

	All patients	Female	Male	Female vs. Male
Total number	n = 1200^a^	n = 1060	n = 139	female: male = 7.63:1
Age of onset (mean years ± SD)	57 ± 12	57 ± 12	60 ± 11	ns
Liver biopsy				
Scheuer 0	9/808 (1.1%)	8/714 (1.1%)	1/94 (1.1%)	ns
Scheuer 1	424/808 (52.5%)	376/714 (52.7%)	48/94 (51.1%)	ns
Scheuer 2	223/808 (27.6%)	197/714 (27.6%)	26/94 (27.7%)	ns
Scheuer 3	80/808 (9.9%)	68/714 (9.5%)	12/94 (12.8%)	ns
Scheuer 4	72/808 (8.9%)	65/714 (9.1%)	7/94 (7.4%)	ns
Outcome				
hepatocellular carcinoma	22/1043 (2.1%)	17/922 (1.8%)	**5/121** **(4.1%)**	ns
liver transplantation	112/1199 (9.4%)	101/1060 (9.5%)	12/139 (8.6%)	ns
fatal hepatic failure	8/1199 (0.7%)	5/1060 (0.5%)	3/139 (2.2%)	ns
Clinical stage at latest evaluation				
I	813/1136 (71.6%)	725/1006 (72.1%)	88/130 (67.7%)	ns
II	188/1136 (16.5%)	163/1006 (16.2%)	25/130 (19.2%)	ns
III	135/1136 (11.9%)	118/1006 (11.7%)	17/130 (13.1%)	ns
Concomitant diseases				
Sjögren’s syndrome	177/1043 (17.0%)	**169/922** (**18**.**3%**)	8/121 (6.6%)	OR = 3.17, p = 0.0013
systemic sclerosis^b^	49/1043 (4.7%)	**47/922** **(5.1%)**	2/121 (1.7%)	ns
Hashimoto’s thyroiditis	102/1043 (9.8%)	96/922 (10.4%)	6/121 (5.0%)	ns (OR = 2.23, p = 0.058)
autoimmune hepatitis	82/1043 (7.9%)	76/922 (8.2%)	6/121 (5.0%)	ns
Raynaud’s phenomenon	32/1043 (3.1%)	31/922 (3.4%)	1/121 (0.8%)	ns
rheumatoid arthritis	44/1043 (4.2%)	**43/922** (**4**.**7%**)	1/121 (0.8%)	OR = 5.87, p = 0.0485
Autoantibodies				
AMA	956/1085 (88.1%)	839/960 (87.4%)	**117/125** (**93**.**6%**)	OR = 0.47, p = 0.0439
ANA	752/1042 (72.2%)	**693/926** (**74**.**8%**)	59/116 (50.9%)	OR = 2.87, p = 5.65 × 10^−8^
gp210	331/1134 (29.2%)	290/1003 (28.9%)	41/131 (31.3%)	ns
CENP-B	324/1166 (27.8%)	**302/1031** (**29**.**3%**)	22/135 (16.3%)	OR = 2.13, p = 0.00153
SS-A	178/1166 (15.3%)	**166/1031** (**16**.**1%**)	12/135 (8.9%)	OR = 1.97, p = 0.0285

The prevalence of complications and autoantibodies was compared between female and male patients with PBC; significantly increased number and frequency were highlighted in bold. ns: not significant.

^a^There are three patients whose information about sex, age and clinical symptoms was not available.

^b^Including limited cutaneous systemic sclerosis (also known as CREST).

Concomitant autoimmune disorders were generally more prevalent in female patients. Among them, Sjögren’s syndrome, systemic sclerosis, and rheumatoid arthritis differed significantly in prevalence between female and male patients. The AMA-positive rate was higher in male than female patients (93.6% vs. 87.4%), but other autoantibodies that may be accompanied by autoimmune complications were more prevalent in female patients. Aside from the higher prevalence of PBC and autoimmune diseases in females, there were no gender-based differences in clinical or pathological features, the prevalence of hepatocellular carcinoma, or the levels of antibodies against the nuclear pore complex gp210.

### *HLA* allele carrier status

In the present study, 158 *HLA* alleles (18 *A*, 42 *B*, 38 *DRB1*, 13 *DQA1*, 22 *DQB1*, 5 *DPA1*, and 20 *DPB1*) or allele groups were identified. Among them, 87 alleles (11 *A*, 21 *B*, 20 *DRB1*, 10 *DQA1*, 11 *DQB1*, 3 *DPA1*, and 11 *DPB1*) that were carried by more than 1% of individuals in either patients or controls were identified and the carrier frequencies compared between the two groups (Supplementary Table 1). Forty-four alleles (3 *A*, 7 *B*, 14 *DRB1*, 5 *DQA1*, 9 *DQB1*, 2 *DPA1*, and 4 *DPB1*) demonstrated positive associations with p values less than 0.05 (Supplementary Table 1). The associations of the 22 remained significant (p < 5.75 × 10^−4^) after correction for multiple-testing (Table 2), and 10 (A*33:03, B*44:03, DRB1*13:02, DRB1*08:03, DQA1*01:02, DQB1*06:04, DQB1*03:01, DQB1*06:01, DPA1*01:03, and DPB1*04:01) reached nominal genome-wide significance (p < 5 × 10^−8^). Although this highly significant association was detected for all six *HLA* loci, the *HLA- DR* and *-DQ* alleles exhibited the most significant effects in terms of both disease-promoting (DRB1*08:03 and DQB1*06:01) and disease-suppressive (DRB1*13:02, DQB1*06:04, and DQB1*03:01) activity.

**Table 2:** Carrier frequencies for selected *HLA* alleles in PBC patients and controls.

*HLA* allele	Carriers in PBC		Carriers in controls		Odds ratio (95% CI)	p value
*HLA-A*		n = 1200		n = 1196		
A*33:03 ^†^	88	(7.3%)	199	(16.6%)	0.40 (0.30–0.52)	2.35 × 10^−12^
A*02:01/07/18	395	(32.9%)	293	(24.5%)	1.51 (1.26–1.81)	5.29 × 10^−6^
*HLA-B*		n = 1200		n = 1196		
B*44:03 ^a^	66	(5.5%)	187	(15.6%)	0.31 (0.23–0.42)	7.01 × 10^−16^
B*07:02	90	(7.5%)	146	(12.2%)	0.58 (0.44–0.77)	1.11 × 10^−4^
*HLA-DRB1*		n = 1200		n = 1194		
DRB1*13:02 ^a^	47	(3.9%)	175	(14.7%)	0.24 (0.17–0.33)	1.35 × 10^−19^
DRB1*08:03 ^a^	283	(23.6%)	179	(15.0%)	1.75 (1.42–2.16)	1.01 × 10^−7^
DRB1*14:03	7	(0.6%)	32	(2.7%)	0.21 (0.09–0.49)	5.10 × 10^−5^
DRB1*04:05	390	(32.5%)	292	(24.5%)	1.49 (1.24–1.78)	1.31 × 10^−5^
*HLA-DQA1*		n = 1198		n = 783		
DQA1*01:02 ^a^	173	(14.4%)	208	(26.6%)	0.47 (0.37–0.59)	2.20 × 10^−11^
*HLA-DQB1*		n = 1199		n = 1195		
DQB1*06:04 ^a^	37	(3.1%)	171	(14.3%)	0.19 (0.13–0.28)	1.91 × 10^−22^
DQB1*03:01 ^a^	144	(12.0%)	256	(21.4%)	0.50 (0.40–0.63)	6.76 × 10^−10^
DQB1*06:01 ^a^	520	(43.4%)	403	(33.7%)	1.51 (1.27–1.78)	1.25 × 10^−6^
DQB1*04:01	378	(31.5%)	280	(23.4%)	1.50 (1.25–1.80)	9.20 × 10^−6^
DQB1*04:02	137	(11.4%)	87	(7.3%)	1.64 (1.24–2.18)	4.99 × 10^−4^
*HLA-DPA1*		n = 1200		n = 783		
DPA1*01:03 ^a^	585	(48.8%)	495	(63.2%)	0.55 (0.46–0.67)	2.58 × 10^−10^
*HLA-DPB1*		n = 1200		n = 1196		
DPB1*04:01 ^a^	35	(2.9%)	131	(11.0%)	0.24 (0.17–0.36)	9.63 × 10^−15^
DPB1*02:01	378	(31.5%)	485	(40.6%)	0.67 (0.57–0.80)	3.95 × 10^−6^
DPB1*05:01	815	(67.9%)	729	(61.0%)	1.36 (1.15–1.60)	3.72 × 10^−4^

The statistical tests of *HLA* alleles were listed for p values less than the significance levels corrected by Bonferroni’s procedure based on the number of the observed alleles in greater than 1% either patients or controls: 11 *A*, 21 *B*, 20 *DRB1*, 10 *DQA1*, 11 *DQB1*, 3 *DPA1* and 11 *DPB1* alleles; total 87 *HLA* alleles; p < 0.05/87 = 5.75 × 10^−4^.

^a^These alleles reached genome-wide significance, p < 5 × 10^−8^.

### *HLA* haplotype analysis

Because the linkage between certain *HLA* alleles is so tight, high level of linkage disequilibrium (LD) occurs, carrying a certain portion of the significant difference in allele or carrier frequencies at the loci of interest observed in patients-control comparisons to potentially be attributable to over- or under-representation of the alleles of other loci, which are more likely to be causative variants. We tried to identify such primary associations among the significant *DRB1* and *DQB1* alleles by haplotype analysis of the four most significant *DRB1-DQB1* combinations (Table 3). The risk conferred by the DRB1*08:03-DQB1*06:01 haplotype (OR = 1.86, p = 1.98 × 10^−9^), but not by haplotypes composed of the other *DRB1* alleles and DQB1*06:01 (OR = 1.11, p = 0.28) indicated that DQB1*06:01 itself had a nominal practical effect on the disease development of PBC (Table 3(A)). Instead, DRB1*08:03 appeared to be primarily associated with the risk of PBC, although the effect of DRB1*08:03 alone could not be evaluated sufficiently because the haplotypes without DQB1*06:01 were observed in only a small number (four persons each) of patients and controls (Table 3(A)). In the case of the combination of DRB1*13:02 and DQB1*06:04, it is likely that DQB1*06:04 is the principal contributor to the disease resistance because the DRB1*13:02-DQB1*06:04 haplotype effect (OR = 0.19, p = 1.15 × 10^−23^, Table 3(B)) was equivalent to the effect of DQB1*06:04 (OR = 0.19, p = 1.15 × 10^−23^, Table 2) and was stronger than that of DRB1*13:02 (OR = 0.24, p = 6.19 × 10^−21^, Table 2). The frequencies of haplotypes including DRB1*13:02 but not DQB1*06:04 were not decreased in the patient group (OR = 1.50, p = 0.40, Table 3(B)). Indeed, carriers of the second most prevalent haplotype consisting of DRB1*13:02, DRB1*13:02-DQB1*06:09 ^17^, were more represented in the patient group (OR = 1.59, p = 0.37, Supplementary Table 2). Furthermore, the protective alleles of the *HLA-A*, *-B*, and *-DP* loci that reached the genome-wide significance, A*33:03, B*44:03, DPA1*01:03, and DPB1*04:01, were all at a high level of LD with DQB1*06:04, and their protective effects were thus considered to be secondary to those of DQB1*06:04, which appeared to be a principal contributor. Similarly, the protective effect of DQB1*03:01 and the risk effect of DQB1*04:01 were suspected based on a comparison of the effects of haplotype carrier status. Specifically, DQB1*03:01 was shared by several protective haplotypes, including DRB1*14:03-DQB1*03:01 (although we could not evaluate the effect of DRB1*14:03 because all chromosomes with DRB1*14:03 also carry DQB1*03:01 (Table 3(C))). DQB1*04:01 elevated disease risk regardless of the presence or absence of DRB1*04:05 in the case of the combination of DRB1*04:05 and DQB1*04:01, but not vice versa (Table 3(D)).

**Table 3:** Carrier status for *HLA-DRB1-DQB1* haplotypes consisting of risk/protective alleles in PBC patients and controls.

*DRB1-DQB1* haplotype	PBC (n = 1199)		Controls (n = 1193)		Odds ratio (95% CI)	p value
(A) DRB1*08:03 and/or DQB1*06:01						
DRB1*08:03-DQB1*06:01	278	(23.2%)	174	(14.6%)	**1**.**77** (**1**.**43–2**.**18**)	**7**.**82 × 10** ^−**8**^
DRB1*08:03-not (DQB1*06:01)	4	(0.3%)	4	(0.3%)	1.33 (0.42–4.21)	0.62
not (DRB1*08:03)-DQB1*06:01	242	(20.2%)	228	(19.1%)	1.11 (0.92–1.34)	0.28
(B) DRB1*13:02 and/or DQB1*06:04						
DRB1*13:02-DQB1*06:04	36	(3.0%)	169	(14.2%)	**0**.**19** (**0**.**13–0**.**27**)	**1**.**84 × 10** ^−**22**^
DRB1*13:02- not (DQB1*06:04)	11	(0.9%)	6	(0.6%)	1.50 (0.58–3.88)	0.40
not (DRB1*13:02)-DQB1*06:04	1	(0.1%)	2	(0.2%)	0.47 (0.04–5.24)	0.53
(C) DRB1*14:03 and/or DQB1*03:01						
DRB1*14:03-DQB1*03:01	7	(0.6%)	32	(2.7%)	**0**.**21** (**0**.**09–0**.**49**)	**5**.**10 × 10** ^−**5**^
DRB1*14:03- not (DQB1*03:01)	0	(0.0%)	0	(0.0%)	—	—
not (DRB1*14:03)-DQB1*03:01	137	(11.4%)	224	(18.8%)	**0**.**54** (**0**.**43–0**.**68**)	**8**.**54 × 10** ^−**8**^
(D) DRB1*04:05 and/or DQB1*04:01						
DRB1*04:05-DQB1*04:01	373	(31.1%)	277	(23.2%)	**1**.**49** (**1**.**24–1**.**79**)	**1**.**44 × 10** ^−**5**^
DRB1*04:05- not (DQB1*04:01)	17	(1.4%)	15	(1.3%)	0.99 (0.55–1.79)	0.98
not (DRB1*04:05)-DQB1*04:01	5	(0.4%)	2	(0.2%)	1.59 (0.38–6.65)	0.53

Haplotypes composed of given *DRB1* and/or *DQB1* alleles were compared. The statistical tests reaching significance (p < 0.05) were highlighted in bold.

### Interaction between *DR-DQ* risk/protective factors

The effect of a given risk/protective factor may enhance or attenuate the action of a second factor beyond the extent anticipated by an independent additive effect model. The interactions between the four most significant primary risk/protective factors identified above were analyzed. For this purpose, augmentation or attenuation of the effect was evaluated by comparing the frequencies of carriers of the factor of interest in subpopulations stratified by the presence or absence of each of the other three factors (Table 4); for example, the effect of DRB1*08:03 was not influenced by the existence of DQB1*03:01 or DQB1*04:01, but was profoundly affected by the presence of DQB1*06:04 (Table 4). It is of noteworthy that the interaction between DRB1*08:03 and DQB1*06:04 was asymmetrical; the protective effect of DQB1*06:04 was not influenced by the disease-promoting effect of DRB1*08:03 (OR = 0.14, p = 0.00417), while the disease-promoting effect of DRB1*08:03 was almost completely negated by the presence of DQB1*06:04 (OR = 1.08, p = 0.92). A similar but inverse asymmetric interaction between DRB1*08:03 and DQB1*03:01 was also demonstrated by stratification analysis; the risk of DRB1*08:03 was evident in the presence of DQB1*03:01 (OR = 2.71, p = 0.000882) but the protective effect conferred by DQB1*03:01 was not observed in the presence of DRB1*08:03 (OR = 0.78, p = 0.41).

**Table 4:** Effect of the risk/protective *DRB1/DQB1* alleles on the presence/absence of another risk/protective *DRB1/DQB1* allele.

Effect of *HLA* allele	*HLA* background	
	Present	Absent
Effect of DRB1*08:03 in the presence/absence of DQB1*06:04		
Frequencies in subset of patients/controls	5.4%/4.7%	**24**.**1%/16**.**6%**
Odds ratio (95% CI), p	1.16 (0.24–5.74), p = 0.85	**1**.**59** (**1**.**29–1**.**97**), **p = 1**.**70 × 10** ^−**5**^
Effect of DQB1*06:04 in the presence/absence of DRB1*08:03		
Frequencies in subset of patients/controls	**0**.**7%/4**.**5%**	**3**.**8%/16**.**1%**
Odds ratio (95% CI), p	**0**.**15** (**0**.**03–0**.**73**), **p = 0**.**0068**	**0**.**21** (**0**.**14–0**.**30**), **p = 8**.**16 × 10** ^−**19**^

The effect of the most significant risk/protective *DRB1* and *DQB1* alleles was evaluated by comparing the frequencies between patients with PBC and control population in the presence/absence of another risk/protective allele. Significant differences (p < 0.05) in the comparisons are highlighted in bold.

### SNP association

**Figure 1:**
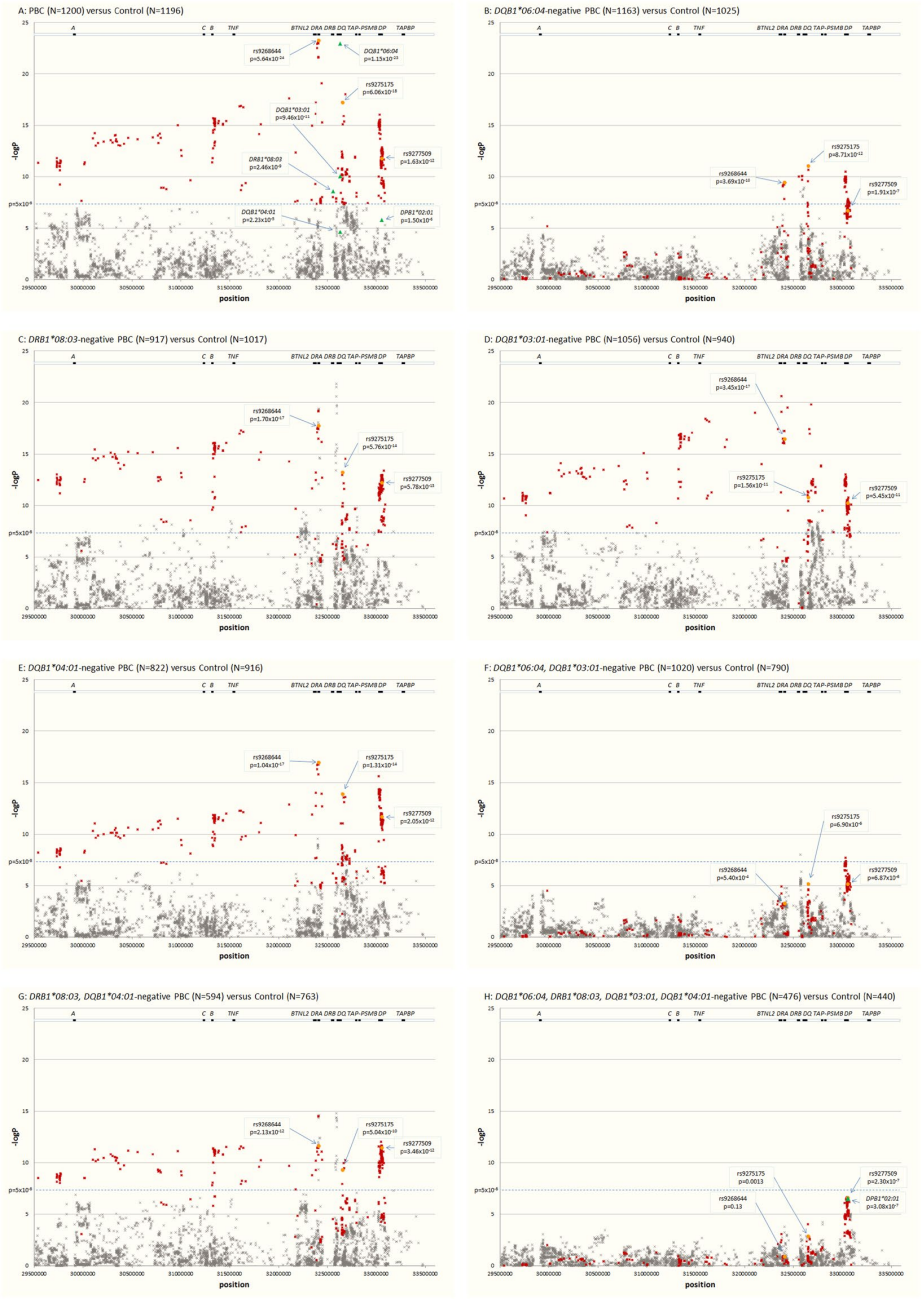
Association signals of genetic markers in the *HLA* region. For each genetic marker -log p was plotted, selected HLA class II alleles (green triangle) and 4103 SNPs (x) in the HLA region (bound by rs446198 at position chr 6:29507426 of GRCh37 assembly and rs367408 at position chr 6:33505746). Three hundred and one SNPs with a p value less than 5 × 10^−8^ (−log p greater than 7.301, the level is shown by blue dotted line) in the comparison between all patients (N = 1200) and controls (N = 1196) are shown by red x symbols throughout the panels. Three SNPs presented in Table 5 are highlighted by orange circles. (**A**) Comparison between all patients and controls; (**B**) DQB1*06:04-negative patients and controls; (**C**) DRB1*08:03-negative patients and controls; (**D**) DQB1*03:01-negative patients and controls; (**E**) DQB1*04:01-negative patients and controls; (**F**) DQB1*06:04-negative, DQB1*03:01-negative patients and controls; (**G**) DRB1*08:03-negative, DQB1*04:01-negative patients and controls; (**H**) all four allele-negative patients and controls. The location of genes encoded in the region, *HLA-A* (*A*), *-B* (*B*), *-C* (*C*), *-DRA1* (*DRA*), *-DRB1* (*DRB*), *-DQA1/-DQB1* (*DQ*), *-DPA1/-DPB1* (*DP*), *TNF*, *BTNL2*, *TAP1/TAP2/PSMB8/PSMB9* (*TAP-PSMB*) and *TAPBP*, is presented at the top of each panel.

For all patients and controls, 4103 SNPs in the *HLA* region (bound by rs446198 at position chr 6:29507426 of GRCh37 assembly and rs367408 at position chr 6:33505746) were genotyped^16, 18^ and the data were further analyzed for the 1200 patients with PBC and 1196 controls whose HLA data were available. When the smaller of two p values which were obtained by applying the dominant effect model of either the predominant allele or the less frequent allele was taken as the effect of each SNP locus, 305 SNPs of them showed p values less than 5 × 10^−8^ (Fig. 1). Among them, rs9268644 near the *HLA-DRA* locus gave the minimal p value (p = 5.64 × 10^−24^) with an odds ratio of 0.39 (Table 5). In our first round of GWAS, rs9275175 in the *HLA-DQB1* locus was identified as the most significant SNP^16^, but it was not as significant as rs9268644 in this setting (OR = 0.41, p = 6.06 × 10^−18^, Table 5). Three hundred and five nominally significant association signals were distributed from the *HLA-A* to *HLA-DP* loci in accordance with the results of the *HLA* association analysis (Fig. 1A).

**Table 5:** Association of SNPs in the *HLA* region with PBC in the absence of major *HLA* factors identified in this study.

Population	Number of individuals		SNPs with p < 5 × 10^−8^	Odds ratio (95% CI), p		
	Patients	Controls		rs9268644^*^	rs9275175^†^	rs9277509^§^
All						
	1200	1196	305/4103	**0**.**39** (**0**.**33–0**.**47**), **p = 5**.**64 × 10** ^−**24**^	0.41 (0.33–0.50), p = 6.06 × 10^−18^	0.55 (0.46–0.65), p = 1.63 × 10^−12^
DQB1*06:04 –negative						
	1163	1025	62/4103	0.52 (0.43–0.64), p = 3.69 × 10^−10^	0.46 (0.36–0.58), p = 8.71 × 10^−12^	0.63 (0.53–0.75), p = 1.91 × 10^−7^
DRB1*08:03 –negative						
	917	1017	361/4103	0.42 (0.34–0.51), p = 1.70 × 10^−18^	0.44 (0.36–0.55), p = 5.76 × 10^−14^	0.51 (0.42–0.61), p = 5.78 × 10^−13^
DQB1*03:01 –negative						
	1056	940	333/4103	0.41 (0.33–0.51), p = 3.45 × 10^−17^	0.43 (0.34–0.56), p = 1.56 × 10^−11^	0.54 (0.45–0.65), p = 5.45 × 10^−11^
DQB1*04:01 –negative						
	822	916	251/4103	0.41 (0.33–0.50), p = 1.04 × 10^−17^	0.43 (0.34–0.53), p = 1.31 × 10^−14^	0.49 (0.40–0.60), p = 2.05 × 10^−12^
DQB1*06:04 -negative, DQB1*03:01 –negative						
	1020	790	3/4103	0.65 (0.51–0.83), p = 0.00054	0.54 (0.41–0.71), p = 6.90 × 10^−6^	0.65 (0.53–0.78), p = 6.87 × 10^−6^
DRB1*08:03 -negative, DQB1*04:01 –negative						
	594	763	225/4103	0.45 (0.35–0.56), p = 2.13 × 10^−12^	0.48 (0.38–0.61), p = 5.04 × 10^−10^	0.45 (0.36–0.57), p = 3.46 × 10^−12^
DQB1*06:04 -negative, DRB1*08:03 -negative, DQB1*03:01 -negative, DQB1*04:01 -negative						
	476	440	0/4103	0.79 (0.59–1.07), p = 0.13	0.62 (0.46–0.83), p = 0.0013	**0**.**49** (**0**.**37–0**.**65**), **p = 2**.**30 × 10** ^−**7**^

*A SNP located in the *DR* locus (the first intron of DRA, chr 6:32408044–32408044), which showed the minimal p value for the dominant model of variant allele in the comparison of all patients and controls (shown in bold).

^†^A SNP located near the *DQ* locus (20 kb upstream of *DQB1*, chr 6: 32654147–32654147), which showed the minimal p value in our previous GWAS (16).

^§^A SNP located in the *DP* locus (the fifth intron of *DPB1*, chr 6: 33054207–33054207), which showed the minimal p value for the dominant model of a variant allele in the absence of four major HLA factors (shown in bold).

To evaluate the contribution of *HLA* alleles on the association signal of these SNPs in the *HLA* region, individuals carrying the *HLA* of interest were excluded from the dominant effect models. We then calculated odds ratio and p value as described above and compared the p values before and after the stepwise exclusion of *HLA* alleles of interest (Table 5 and Fig. 1B–H). Among the four most significantly associated *HLA* alleles, DQB1*06:04 shows strongest impact as revealed by the comparison between 1163 DQB1*06:04-negative patients and 1025 DQB1*06:04-negative controls in which only 62 SNPs remained with a p value less than 5 × 10^−8^ (Table 5 and Fig. 1B), although the other three alleles did as well but to a reduced extent (Fig. 1C–E). Furthermore, when the two protective alleles DQB1*06:04 and DQB1*03:01 were combined, 302 signals became less significant than the threshold (Fig. 1F), while the combination of two disease-promoting alleles, DRB1*08:03 and DQB1*04:01 had a weaker impact (Fig. 1G) even though the number of patients and controls remained larger than the case that for the protective alleles. In the absence of the four *HLA-DR/DQ* alleles, no SNP reached a nominal genome-wide significance level, but a peak association signal was found in the *HLA-DPB1* locus, rs9277509 with p = 2.30 × 10^−7^ and an odds ratio of 0.49 in the comparison of the four *HLA-DR/DQ*-negative subpopulation of patients (N = 476) and controls (N = 440) (Table 5 and Fig. 1H). The fact that the SNP association signal remained after excluding the carriers of these four *HLA-DR/DQ* alleles could be explained by DPB1*02:01 exhibiting a similar p value (3.08 × 10^−7^) to the same subpopulation analysis (Fig. 1H).

## Discussion

The association of certain SNP alleles in the *HLA* class II region with the development of PBC has been consistently reported as a major finding in several GWAS studies across different ethnic groups, including Japanese^14–16^. These results suggest that one or more *HLA* class II–linked genetic factors influence susceptibility to PBC, a theory that has been strongly supported by a number of HLA association studies^8–13^. In the present study, we recruited a large number of patients and healthy control individuals and could therefore obtain confirmatory results at genome-wide significance levels (p < 5 × 10^−8^) for the strongest disease-promoting effect of DRB1*08:03 and the strongest protective effect of DQB1*06:04 in Japanese individuals. The effects of both haplotypes were previously reported by us^10^ and others^12^. Further, 22 out of the 87 *HLA* factors examined in the present study were significant after Bonferroni’s correction for multiple testing (p < 5.75 × 10^−4^), which is known to be very conservative. This was the case even when we applied the statistical test for the comparison of carrier frequencies, which is generally less sensitive than the test for allele frequencies but is more relevant for the dominant model of inheritance. Because of LD between the alleles carried by common *HLA* haplotypes in the ethnic population of interest, some of our findings result from secondary associations due to LD with the primary allele. Therefore, we performed haplotype association analysis to discern possible interdependencies among them. We identified four major risk/protective factors in the *HLA-DR-DQ* region for PBC in a Japanese population, DQB1*06:04, DRB1*08:03, DQB1*03:01, and DQB1*04:01. As discussed in previous reports^8, 9, 11^, some of these risk/protective *HLA* alleles share similarities with clinically important alleles in other populations, such as the risk-increasing DRB1*08:01 allele and the protective DRB1*11 and DRB1*13 alleles in individuals of European descent. As shown in previous studies, the molecular basis underlying the effects of alleles, whether they are risk-promoting or protective, may involve common amino acid residues exclusive to each group^8, 12^. However, false association errors hindered the results of these studies, and therefore determining the principally associated allele is critical for this type of analysis.

The identification of multiple independent risk/protective factors within the *HLA* region prompted us to evaluate the effects of an interaction between them, since this has not previously been performed except for a study examining the genotype effect of the *HLA-DRB1* locus^9^, in which the risk-promoting effect of DRB1*08 and the protective effect of DRB1*11 were independent and competed with each other. Some risk/protective factors behaved differently, being either enhanced or attenuated, depending on the presence or absence of another factor. For example, the major disease-promoting effect associated with DRB1*08:03 disappeared almost completely in the presence of the protective effect of DQB1*06:04. A similar but opposite relationship between the disease-promoting *HLA-DR* and the protective *HLA-DQ* factors was observed between DRB1*08:03 and DQB1*03:01. Recently, DQB1*06:04 (and DRB1*13:02) was also reported to be a protective allele against autoimmune thyroid diseases^19^; this allele exhibited dominant epistatic effects on *HLA* risk factors in a similar fashion to that observed in our study. Elucidating the immunological implications of the unrivaled protective effect conferred by DQB1*06:04 may lead to the identification of an active suppressive mechanism for the development of PBC, and could thus identify potential targets for disease prevention.

Epigenetic control of gene expression is another factor that could further elucidate the genetic contribution for PBC disease susceptibility which was not covered by studies of genetic polymorphisms such as our *HLA* analysis or GWAS. Indeed, alterations in DNA methylation patterns in immune cells were found in patients with PBC^20, 21^. Furthermore, somatic changes in the genetic material such as sex chromosome loss leading to monosomy X were also reported and may elucidate the underlying mechanism for the female predominance of PBC^22, 23^.

In summary, this study analyzed a large population of patients with PBC and an equivalently sized control group to confirm the presence of multiple disease-promoting and -protective genetic factors in the *HLA* region. Interactions between these genetic factors will provide a better understanding of the complicated pathogenic mechanisms of PBC.

## Methods

### Study design

Case-control study: patient samples were collected at 60 medical institutions in Japan, 32 of which belong to the National Hospital Organization Study Group for Liver Disease in Japan (NHOSLJ). After obtaining written informed consent, we collected patient blood samples for serum and DNA analysis. All study protocols were approved by the institutional review boards of Nagasaki University, NHOSLJ and the other participating institutions according to the Declaration of Helsinki issued by the World Medical Association.

### Subject population

All patients met at least two of the following three criteria for the definitive diagnosis of PBC: (i) persistent elevation of serum alkaline phosphatase, an enzyme indicative of cholestasis; (ii) positive AMA test; and (iii) liver biopsy showing non-suppurative inflammation and destruction of the interlobular bile ducts (florid duct lesions), which are characteristics of PBC^3^. Patients with positive serological markers for persistent hepatitis B or C virus infection were excluded from this study. Liver biopsy data were available for 857 of the 1280 patients (67.0%). Histological diagnosis and staging was performed according to Scheuer’s classification^3^. Patients were categorized into three different clinical stages based on liver biopsy results and clinical manifestations: clinical stage I, Scheuer’s stage 1 or 2 in liver biopsy or unknown histological stage without signs of portal hypertension or liver cirrhosis; clinical stage II, Scheuer’s stage 3 or 4 in liver biopsy or any histological stage with signs of portal hypertension or liver cirrhosis but without jaundice (total bilirubin less than 2 mg/dL); clinical stage III, any Scheuer’s stage with persistent jaundice (total bilirubin 2 mg/dL or above). Data for clinical staging were provided by patients’ primary caregivers via the collection of fixed case record form.

### *HLA* genotyping

The *HLA-A*, *-B*, *-DQA1*, and *-DQB1* genotypes were determined by HLA-DNA typing kits based on reverse SSO hybridization using Luminex xMAP technology, LABType SSO® (One Lambda, Canoga Park, CA, USA) and WakFlow HLA (Wakunaga Pharmaceutical, Osaka, Japan) according to the manufacturers’ instructions. The *HLA-DRB1*, *-DPA1*, and *-DPB1* genotypes were determined by direct sequencing of the PCR products with 3730 Genetic Analyzer (Applied Biosystems, Foster City, CA, USA), followed by matching to an allele database implemented in ASSIGN ATF ver. 1.0.2.45. as described elsewhere^20^. Most *HLA* alleles were genotyped at the four-digit level, which correspond to the unique amino acid sequences of the precursor polypeptides. However, because of ambiguous matching of probe reaction patterns in HLA genotyping, the following groups of alleles could not be distinguished in certain individuals and the four-digit designation could therefore not be achieved: a group of A*02 alleles with Phe at position 9 (A*02:01, A*02:07, and A*02:18), another A*02 allele group with Tyr at position 9 (A*02:06 and A*02:10), A*11 alleles (A*11:01 and A*11:02), A*24 alleles (A*24:02 and A*24:20), B*13 alleles (B*13:01 and B*13:02), B*15 alleles with B62 antigen specificity (B*15:01, B*15:07, B*15:27, and B*15:28), B*15 alleles with B75 antigen specificity (B*15:02 and B*15:11), and DQA1*03 alleles (DQA1*03:01, DQA1*03:02, and DQA1*03:03). These alleles were designated A*02:01/07/18, A*02:06/10, A*11, A*24, B*13, B*15:01/07/27/28, B*15:02/11, and DQA1*03, respectively. Certain *DRB1*, *DPA1*, and *DPB1* alleles were not distinguished from others if the nucleotide sequences of the second exon were indistinguishable, e.g. DRB1*12:01, DRB1*12:06, and DRB1*12:10. In this case, the most probable allele, DRB1*12:01, was assigned.

### SNP genotyping

The study population in the present study includes 487 patients and 476 controls who were analyzed in our first round of GWAS^16^. Other patients and controls were collected for the second phase of GWAS^18^. After collection and cleaning of SNP genotyping data as previously described^16^, the genotype data for 4103 SNPs in the HLA region (bounded by rs446198 at position chr 6:29507426 of GRCh37 assembly and rs367408 at position chr 6:33505746) were evaluated. The subjects’ first, second and third-degree relatives (parent-offspring, siblings, uncle/aunt-nephew/niece) were excluded from this study based on a test of identity-by-descent using SNP data collected in the GWAS.

### Statistical analysis

The association of disease phenotype and *HLA* carrier status or SNP genotype was evaluated by the odds ratio as calculated by Woolf’s formula and examined by the chi-square test with 2 × 2 contingency tables, unless otherwise indicated. *HLA-DRB1-DQB1* haplotypes were empirically determined for all genotyped individuals with reference to publically accessible HLA haplotype frequency data (HLA Laboratory, Kyoto Japan, http://hla.or.jp/haplo/haplodl.php?lang = en). Haplotype association analysis was applied to compare the relative effect size between *HLA-DRB1* and *-DQB1* alleles consisting of significant haplotypes. In order to examine the interaction between selected HLA alleles, the effect of HLA carrier status was evaluated in subpopulations stratified by another HLA carrier status of interest. Statistical tests, including those mentioned above, were performed using STATA Release 12 (StataCorp, College Station, TX, USA).

## Electronic supplementary material

supplementary tables
