# Supplementary material for: Principal contribution of HLA-DQ alleles, DQB1*06:04 and DQB1*03:01, to disease resistance against primary biliary cholangitis in a Japanese population
Source: Sci Rep. 2017 Sep 11;7:11093. doi: 10.1038/s41598-017-11148-6 (PMC5593890; doi:10.1038/s41598-017-11148-6)
Supplement: Supplementary file 1 — supplementary tables [file 41598_2017_11148_MOESM1_ESM.doc]

Supplementary Information

**Principal contribution of *HLA-DQ* alleles, *DQB1*06:04* and *DQB1*03:01*, to disease resistance against primary biliary cholangitis in a Japanese population**

Authors:

Michio Yasunami^1,2^*, Hitomi Nakamura^2,3^, Katsushi Tokunaga^4^, Minae Kawashima^4^, Nao Nishida^4,5^, Yuki Hitomi^4^, Minoru Nakamura^3,6,7^*

Affiliations:

^1^Department of Medical Genomics, Life Science Institute, Saga-Ken Medical Centre Koseikan, Saga, 840-8571, Japan

^2^Department of Clinical Medicine, Institute of Tropical Medicine, Nagasaki University, Nagasaki, 852-8523, Japan

^3^Clinical Research Center, National Hospital Organization (NHO) Nagasaki Medical Center and Department of Hepatology, Nagasaki University Graduate School of Biomedical Sciences, Omura, 856-8562, Japan

^4^Department of Human Genetics, Graduate School of Medicine, the University of Tokyo, Tokyo, 113-0033, Japan

^5^The Research Center for Hepatitis and Immunology, National Center for Global Health and Medicine, Ichikawa 272-8516, Japan

^6^Headquarters of PBC Research in the NHO Study Group for Liver Disease in Japan (NHOSLJ)

^7^Headquarters of gp210 Working Group in Intractable Hepatobiliary Disease Study Group supported by the Ministry of Health, Labour and Welfare of Japan (gp210WG)

*Co-corresponding authors:

e-mail: yasunami-michio@koseikan.jp (M. Y.)

e-mail: nakamuram@nagasaki-mc.com (M. N.)

Supplementary Table 1. Carrier frequencies for *HLA* alleles in PBC patients and controls

| *HLA* allele | carriers in PBC | | carriers in controls | | odds ratio (95% CI) | p value |
| --- | --- | --- | --- | --- | --- | --- |
| *HLA-A* |  | n=1200 |  | n=1196 |  |  |
| *A*01:01* | 8 | (0.7%) | 14 | (1.2%) |  |  |
| *A*02:01/07/18* | 395 | (32.9%) | 293 | (24.5%) | 1.51 (1.26-1.81) | 5.29 x 10^-6^ |
| *A*02:06/10* | 224 | (20.3%) | 188 | (15.7%) | 1.37 (1.11-1.69) | 0.00331 |
| *A*03:01* | 9 | (0.8%) | 12 | (1.0%) |  |  |
| *A*11* alleles | 244 | (18.7%) | 214 | (17.9%) |  |  |
| *A*24* alleles | 714 | (59.5%) | 739 | (61.8%) |  |  |
| *A*26:01* | 160 | (13.3%) | 179 | (15.0%) |  |  |
| *A*26:02* | 42 | (3.5%) | 50 | (4.2%) |  |  |
| *A*26:03* | 63 | (5.3%) | 57 | (4.8%) |  |  |
| *A*31:01* | 177 | (14.8%) | 169 | (14.1%) |  |  |
| *A*33:03* | 88 | (7.3%) | 199 | (16.6%) | 0.40 (0.30-0.52) | 2.35 x 10^-12^ |
| *HLA-B* |  | n=1200 |  | n=1196 |  |  |
| *B*07:02* | 90 | (7.5%) | 146 | (12.2%) | 0.58 (0.44-0.77) | 1.11 x 10^-4^ |
| *B*13* alleles | 16 | (1.3%) | 29 | (2.4%) | 0.54 (0.29-1.01) | 0.0492 |
| *B*15:01/07/27/28* | 144 | (12.0%) | 202 | (16.9%) | 0.67 (0.53-0.85) | 6.63 x 10^-4^ |
| *B*15:02/11* | 24 | (2.0%) | 18 | (1.5%) |  |  |
| *B*15:18* | 17 | (1.4%) | 37 | (3.1%) | 0.45 (0.25-0.80) | 0.00571 |
| *B*35:01* | 196 | (16.3%) | 157 | (13.1%) | 1.29(1.03-1.62) | 0.00269 |
| *B*37:01* | 13 | (1.1%) | 11 | (0.9%) |  |  |
| *B*39:01* | 94 | (7.8%) | 89 | (7.4%) |  |  |
| *B*40:01* | 149 | (12.4%) | 119 | (9.9%) | 1.28 (0.99-1.66) | 0.0554 |
| *B*40:02/03/06* | 343 | (28.6%) | 292 | (24.4%) | 1.24(1.03-1.49) | 0.0208 |
| *B*44:03* | 66 | (5.5%) | 187 | (15.6%) | 0.31 (0.23-0.42) | 7.01 x 10^-16^ |
| *B*46:01* | 164 | (13.7%) | 116 | (9.7%) | 1.47 (1.14-1.90) | 0.00251 |
| *B*48:01* | 62 | (5.2%) | 68 | (5.7%) |  |  |
| *B*51:01* | 184 | (15.3%) | 183 | (15.3%) |  |  |
| *B*52:01* | 268 | (22.3%) | 270 | (22.6%) |  |  |
| *B*54:01* | 194 | (16.2%) | 174 | (14.5%) |  |  |
| *B*55:02* | 70 | (5.8%) | 57 | (4.8%) |  |  |
| *B*56:01* | 23 | (1.9%) | 22 | (1.8%) |  |  |
| *B*58:01* | 14 | (1.2%) | 10 | (0.8%) |  |  |
| *B*59:01* | 47 | (3.9%) | 46 | (3.8%) |  |  |
| *B*67:01* | 34 | (2.8%) | 26 | (2.2%) |  |  |
| *HLA-DRB1* |  | n=1200 |  | n=1194 |  |  |
| *DRB1*01:01* | 101 | (8.4%) | 146 | (12.2%) | 0.66 (0.50-0.86) | 0.00218 |
| *DRB1*04:01* | 21 | (1.8%) | 26 | (2.2%) |  |  |
| *DRB1*04:03* | 61 | (5.1%) | 50 | (4.2%) |  |  |
| *DRB1*04:05* | 390 | (32.5%) | 292 | (24.5%) | 1.49 (1.24-1.78) | 1.31 x 10^-5^ |
| *DRB1*04:06* | 49 | (4.1%) | 78 | (6.5%) | 0.61 (0.42-0.88) | 0.00754 |
| *DRB1*04:10* | 55 | (4.6%) | 37 | (3.1%) | 1.50 (0.98-2.30) | 0.0588 |
| *DRB1*08:02* | 123 | (10.3%) | 98 | (8.2%) | 1.28 (0.97-1.69) | 0.0843 |
| *DRB1*08:03* | 283 | (23.6%) | 179 | (15.0%) | 1.75 (1.42-2.16) | 1.01 x 10^-7^ |
| *DRB1*09:01* | 313 | (26.1%) | 321 | (26.9%) |  |  |
| *DRB1*11:01* | 30 | (2.5%) | 58 | (4.9%) | 0.50 (0.32-0.79) | 0.00218 |
| *DRB1*12:01* | 46 | (3.8%) | 81 | (6.8%) | 0.55 (0.38-0.79) | 0.00128 |
| *DRB1*12:02* | 22 | (1.8%) | 46 | (3.9%) | 0.47 (0.28-0.78) | 0.00295 |
| *DRB1*13:02* | 47 | (3.9%) | 175 | (14.7%) | 0.24 (0.17-0.33) | 1.35x 10^-19^ |
| *DRB1*14:03* | 7 | (0.6%) | 32 | (2.7%) | 0.21 (0.09-0.49) | 5.10 x 10^-5^ |
| *DRB1*14:05* | 90 | (7.5%) | 52 | (4.4%) | 1.78 (1.25-2.53) | 0.00113 |
| *DRB1*14:06* | 28 | (2.3%) | 36 | (3.0%) |  |  |
| *DRB1*14:54* | 102 | (8.5%) | 76 | (6.4%) | 1.37(1.00-1.86) | 0.0466 |
| *DRB1*15:01* | 130 | (10.8%) | 171 | (14.3%) | 0.73 (0.57-0.93) | 0.0101 |
| *DRB1*15:02* | 266 | (22.2%) | 254 | (21.3%) |  |  |
| *DRB1*16:02* | 30 | (2.5%) | 14 | (1.2%) | 2.16 (1.14-4.10) | 0.0157 |
| *HLA-DQA1* |  | n=1198 |  | n=783 |  |  |
| *DQA1*01:01* | 296 | (24.7%) | 200 | (25.5%) |  |  |
| *DQA1*01:02* | 173 | (14.4%) | 208 | (26.6%) | 0.47 (0.37-0.59) | 2.20 x 10^-11^ |
| *DQA1*01:03* | 523 | (43.7%) | 286 | (36.5%) | 1.35 (1.12-1.62) | 0.00160 |
| *DQA1*02:01* | 9 | (0.8%) | 8 | (1.0%) |  |  |
| *DQA1*03* alleles | 822 | (73.6%) | 490 | (62.6%) | 1.30 (1.08-1.58) | 0.00617 |
| *DQA1*04:01* | 83 | (6.9%) | 40 | (5.1%) |  |  |
| *DQA1*05:03* | 36 | (3.0%) | 48 | (6.1%) | 0.47 (0.30-0.74) | 7.42 x 10^-4^ |
| *DQA1*05:05* | 55 | (4.6%) | 62 | (7.9%) | 0.56 (0.38-0.81) | 0.00213 |
| *DQA1*05:08* | 8 | (0.7%) | 8 | (1.0%) |  |  |
| *DQA1*06:01* | 30 | (2.5%) | 31 | (4.0%) |  |  |
| *HLA-DQB1* |  | n=1199 |  | n=1195 |  |  |
| *DQB1*03:01* | 144 | (12.0%) | 256 | (21.4%) | 0.50 (0.40-0.63) | 6.76 x 10^-10^ |
| *DQB1*03:02* | 145 | (12.1%) | 162 | (13.6%) |  |  |
| *DQB1*03:03* | 386 | (32.2%) | 382 | (32.0%) |  |  |
| *DQB1*04:01* | 378 | (31.5%) | 280 | (23.4%) | 1.50 (1.25-1.80) | 9.20 x 10^-6^ |
| *DQB1*04:02* | 137 | (11.4%) | 87 | (7.3%) | 1.64 (1.24-2.18) | 4.99 x 10^-4^ |
| *DQB1*05:01* | 114 | (9.5%) | 162 | (13.6%) | 0.67 (0.52-0.86) | 0.00194 |
| *DQB1*05:02* | 84 | (7.0%) | 54 | (4.5%) | 1.59 (1.12-2.26) | 0.00906 |
| *DQB1*05:03* | 138 | (11.5%) | 92 | (7.7%) | 1.56 (1.18-2.06) | 0.00157 |
| *DQB1*06:01* | 520 | (43.4%) | 403 | (33.7%) | 1.51 (1.27-1.78) | 1.25 x 10^-6^ |
| *DQB1*06:02* | 116 | (9.7%) | 169 | (14.1%) | 0.65 (0.51-0.84) | 7.38 x 10^-4^ |
| *DQB1*06:04* | 37 | (3.1%) | 171 | (14.3%) | 0.19 (0.13-0.28) | 1.91x 10^-22^ |
| *HLA-DPA1* |  | n=1200 |  | n=783 |  |  |
| *DPA1*01:03* | 585 | (48.8%) | 495 | (63.2%) | 0.55 (0.46-0.67) | 2.58 x 10^-10^ |
| *DPA1*02:01* | 399 | (33.3%) | 247 | (31.5%) |  |  |
| *DPA1*02:02* | 873 | (72.8%) | 534 | (68.2%) | 1.24 (1.02-1.51) | 0.0324 |
| *HLA-DPB1* |  | n=1200 |  | n=1196 |  |  |
| *DPB1*02:01* | 378 | (31.5%) | 485 | (40.6%) | 0.67 (0.57-0.80) | 3.95 x 10^-6^ |
| *DPB1*02:02* | 109 | (9.1%) | 98 | (8.2%) |  |  |
| *DPB1*03:01* | 126 | (10.5%) | 115 | (9.6%) |  |  |
| *DPB1*04:01* | 35 | (2.9%) | 131 | (11.0%) | 0.24 (0.17-0.36) | 9.63 x 10^-15^ |
| *DPB1*04:02* | 181 | (15.1%) | 226 | (18.9%) | 0.76 (0.62-0.94) | 0.013 |
| *DPB1*05:01* | 815 | (67.9%) | 729 | (61.0%) | 1.36 (1.15-1.60) | 3.72 x10^-4^ |
| *DPB1*06:01* | 15 | (1.3%) | 22 | (1.8%) |  |  |
| *DPB1*09:01* | 256 | (21.3%) | 230 | (19.2%) |  |  |
| *DPB1*13:01* | 42 | (3.5%) | 47 | (3.9%) |  |  |
| *DPB1*14:01* | 41 | (3.4%) | 29 | (2.4%) |  |  |
| *DPB1*19:01* | 14 | (1.2%) | 12 | (1.0%) |  |  |

Supplementary Table 2. Frequencies for *HLA-DRB1-DQB1* haplotypes in PBC patients and controls

| Haplotype | in PBC | | in controls | | OR (95%CI) | p value |
| --- | --- | --- | --- | --- | --- | --- |
| *DRB1*13:02* haplotypes |  |  |  |  |  |  |
| ***DRB1*13:02-DQB1*06:04*** | 40 | (3.1%) | 175 | (14.4%) | **0.19 (0.13-0.28)** | **1.15 x 10^-23^** |
| *DRB1*13:02-DQB1*06:09* | 10 | (0.8%) | 6 | (0.5%) | 1.59 (0.58-4.38) | 0.37 |
| *DQB1*06:01* haplotypes |  |  |  |  |  |  |
| ***DRB1*08:03-DQB1*06:01*** | 308 | (24.1%) | 177 | (14.6%) | **1.86 (1.51-2.29)** | **1.98 x 10^-9^** |
| *DRB1*15:02-DQB1*06:01* | 278 | (21.8%) | 250 | (20.6%) | 1.07 (0.88-1.30) | 0.48 |
| *DQB1*03:01* haplotypes |  |  |  |  |  |  |
| ***DRB1*14:03-DQB1*03:01*** | 7 | (0.5%) | 33 | (2.7%) | **0.20 (0.09-0.45)** | **1.64 x 10^-5^** |
| ***DRB1*11:01-DQB1*03:01*** | 23 | (1.8%) | 53 | (4.4%) | **0.40 (0.24-0.66)** | **1.97 x 10^-4^** |
| ***DRB1*12:02-DQB1*03:01*** | 22 | (1.7%) | 47 | (3.9%) | **0.43 (0.26-0.73)** | **0.00108** |
| ***DRB1*12:01-DQB1*03:01*** | 31 | (2.4%) | 58 | (4.8%) | **0.50 (0.32-0.77)** | **0.00157** |
| *DRB1*15:01-DQB1*03:01* | 12 | (0.9%) | 5 | (0.4%) | 2.29 (0.80-6.53) | 0.11 |
| *DRB1*14:06-DQB1*03:01* | 28 | (2.2%) | 36 | (3.0%) | 0.73 (0.44-1.21) | 0.22 |
| *DRB1*04:01-DQB1*03:01* | 21 | (1.6%) | 23 | (1.9%) | 0.87 (0.48-1.57) | 0.63 |
| *DRB1*04:05* haplotypes |  |  |  |  |  |  |
| ***DRB1*04:05-DQB1*04:01*** | 389 | (30.5%) | 280 | (23.1%) | **1.46 (1.22-1.75)** | **3.31 x 10^-5^** |
| *DRB1*04:05-DQB1*04:02* | 10 | (0.8%) | 7 | (0.6%) | 1.36 (0.52-3.58) | 0.53 |
| *DRB1*08:02* haplotypes |  |  |  |  |  |  |
| ***DRB1*08:02-DQB1*04:02*** | 78 | (6.1%) | 43 | (3.5%) | **1.77 (1.21-2.59)** | **0.00295** |
| ***DRB1*08:02-DQB1*03:03*** | 22 | (1.7%) | 9 | (0.7%) | **2.35 (1.07-5.12)** | **0.0273** |
| *DRB1*08:02-DQB1*03:02* | 41 | (3.2%) | 45 | (3.7%) | 0.86 (0.56-1.32) | 0.50 |
| *DR1* haplotype |  |  |  |  |  |  |
| ***DRB1*01:01-DQB1*05:01*** | 104 | (8.1%) | 147 | (12.1%) | **0.64 (0.49-0.84)** | **9.93 x 10^-4^** |
| *DR2* haplotypes |  |  |  |  |  |  |
| ***DRB1*15:01-DQB1*06:02*** | 121 | (9.5%) | 166 | (13.7%) | **0.66 (0.51-0.85)** | **0.00101** |
| *DRB1*15:01-DQB1*03:01* | 12 | (0.9%) | 5 | (0.4%) | 2.29 (0.80-6.53) | 0.11 |
| ***DRB1*16:02-DQB1*05:02*** | 33 | (2.6%) | 14 | (1.2%) | **2.27 (1.21-4.27)** | **0.00877** |
| *DR4* haplotypes |  |  |  |  |  |  |
| ***DRB1*04:05-DQB1*04:01*** | 389 | (30.5%) | 280 | (23.1%) | **1.46 (1.22-1.75)** | **3.31 x 10^-5^** |
| *DRB1*04:05-DQB1*04:02* | 10 | (0.8%) | 7 | (0.6%) | 1.36 (0.52-3.58) | 0.53 |
| *DRB1*04:10-DQB1*04:02* | 56 | (4.4%) | 36 | (3.0%) | 1.50 (0.98-2.30) | 0.061 |
| ***DRB1*04:06-DQB1*03:02*** | 39 | (3.1%) | 62 | (5.1%) | **0.58 (0.39-0.88)** | **0.00932** |
| *DRB1*04:01-DQB1*03:01* | 21 | (1.6%) | 23 | (1.9%) | 0.87 (0.48-1.57) | 0.63 |
| *DR14* haplotypes |  |  |  |  |  |  |
| ***DRB1*14:05-DQB1*05:03*** | 85 | (6.7%) | 49 | (4.0%) | **1.69 (1.18-2.43)** | **0.00384** |
| ***DRB1*14:03-DQB1*03:01*** | 7 | (0.5%) | 33 | (2.7%) | **0.20 (0.09-0.45)** | **1.64 x 10^-5^** |
| *DRB1*14:06-DQB1*03:01* | 28 | (2.2%) | 36 | (3.0%) | 0.73 (0.44-1.21) | 0.22 |
| *DRB1*14:54-DQB1*05:03* | 54 | (4.2%) | 39 | (3.2%) | 1.33 (0.87-2.02) | 0.18 |
| *DRB1*14:54-DQB1*05:02* | 50 | (3.9%) | 35 | (2.9%) | 1.37 (0.88-2.13) | 0.16 |

The haplotypes consist of given *HLA* alleles are listed under subheadings with *“<HLA allele name>* haplotypes*”*, which were present in more than 0.5% either in patients or in control population. Significant difference (p < 0.05) found in the comparisons are highlighted in **bold**.
